# A randomised controlled trial comparing three clinical administration strategies in spectral detector CT pulmonary angiography with low contrast medium dose

**DOI:** 10.1007/s00330-025-11420-8

**Published:** 2025-02-19

**Authors:** Cathrine Helgestad Kristiansen, Owen Thomas, Anton Bengt Nyquist, Audun Sanderud, Joao Boavida, Jonn Terje Geitung, Thien Trung Tran, Peter Mæhre Lauritzen

**Affiliations:** 1 Department of Life Sciences and Health, Oslo Metropolitan University, Oslo, Norway; 2 Department of Diagnostic Imaging and Intervention, Akershus University Hospital, Lørenskog, Norway; 3 Health Services Research Department (HØKH), Akershus University Hospital, Lørenskog, Norway; 4 Decommissioning Department, Norwegian Nuclear Decommissioning, Halden, Norway; 5 Department of Diagnostic Imaging, Nordland Hospital, Bodø, Norway; 6 Institute of Clinical Medicine, University of Oslo, Oslo, Norway; 7 Division of Radiology and Nuclear Medicine, Oslo University Hospital, Oslo, Norway

**Keywords:** Contrast media, Pulmonary embolism, Image enhancement, Computed tomography angiography, Administration and dosage

## Abstract

**Objectives:**

To compare vascular attenuation (VA) with three strategies for administering a low contrast medium (CM) dose in dual-layer spectral detector CT pulmonary angiography (CTPA).

**Methods:**

Patients were prospectively randomised into control- or one of two experimental groups. Control group patients received CM (350 mgI/mL) diluted 1:1 with saline. Experimental group B received CM (350 mgI/mL) with low flow. Experimental group C received CM with low concentration (140 mgI/mL). Virtual monoenergetic images at 40 and 55 kiloelectron Volt (keV) were reconstructed. Objective examination quality (OEQ) i.e., VA, noise, and signal-to-noise ratio, was measured and subjective examination quality (SEQ) was rated at three anatomical levels: in the pulmonary trunk (PT), the interlobar arteries and the posterior basal segmental arteries. Primary outcome: VA in PT at 40 keV. Secondary outcomes: OEQ and SEQ across all anatomic levels.

**Results:**

A total of 328 patients were randomised. 112 vs 115 and 101 were analysed in the control (A) vs experimental groups (B and C), respectively. There were no differences in VA in PT between the groups: A vs B (*p* = 0.96), B vs C (*p* = 0.14), and A vs C (*p* = 0.18). Group C showed higher VA across all anatomical levels. There were no differences in SEQ.

**Conclusion:**

There was no difference in the attenuation in the PT between the dilution-, low flow-, and low concentration groups. However, the low concentration group showed higher attenuation in the pulmonary arteries when all anatomical levels were assessed.

**Key Points:**

***Question*** *Contrast medium reduction may be accomplished with dilution, low flow, or low concentration. However, the effect of the different strategies on vascular attenuation is unknown*.

***Findings ****There was no difference in pulmonary trunk attenuation between the three strategies on spectral detector CT pulmonary angiography*.

***Clinical relevance**** Low contrast medium dose spectral detector CT pulmonary angiography may be implemented with the administration strategy of the unit’s own choice*.

## Introduction

CT pulmonary angiography (CTPA) is the diagnostic imaging test of choice for pulmonary embolism (PE) [1]. The iodinated contrast medium (CM) dose required for CTPA may be reduced by approximately 50% from a standard dose of approximately 26 g of iodine with three CT techniques: low-kV, subtraction (contrast boost technique), and dual-energy (DE) scanning [2–7].

CM dose reduction may reduce adverse effects, pollution of drinking water, CM shortages and hospital costs [2, 8–12]. Dual-energy CT (DECT) generate high- and low-energy CT data, which allow the reconstruction of virtual monoenergetic images (VMI) at different levels [13]. Recently, supplementary spectral DECT data demonstrated added diagnostic value in PE [14, 15]. The lowest VMI level on most scanners is 40 kiloelectron volts (keV) (roughly equivalent to 70 kilovolt peak (kVp) on single energy scanners), which yields the largest increase in the attenuation of iodine [16]. However, some studies advocate 50 or 55 keV, which has less noise, for the diagnosis of pulmonary embolism [16, 17].

Administration of a reduced CM dose may be accomplished by three different strategies used in clinical practice [18, 19]. In the dilution technique, the injected volume is a 50/50 blend of CM and saline. The low flow technique reduces the total CM dose by injecting the undiluted CM at a lower rate. CM dose may also be reduced by injecting a lower concentration of iodinated CM. In all three techniques, it is recommended to use a saline chaser to keep the CM injection tightly grouped.

Although CTPA is a common examination, it can be a challenge to deliver the expected high diagnostic performance. Specifically, frequent causes of non-diagnostic CTPA are motion artefacts, poor contrast enhancement, timing or transient interruption of contrast inflow into the right heart [20].

To our knowledge no study has compared vascular attenuation (VA) between different strategies for administration of reduced CM in DE CTPA. This study compares three different strategies for administration of reduced CM dose using DE: dilution, low flow, and low concentration.

### Hypothesis

Our hypothesis was that the three strategies for CM administration: dilution, low flow, and low concentration showed no difference in attenuation in the pulmonary trunk (PT).

## Materials and methods

The Regional Committee for Medical and Health Research Ethics of Southeast Norway (142126) and the Data Protection Officer of the hospital approved the study. All participants gave written informed consent. The study was post-date registered on 24 October 2022 at ClinicalTrials.gov (NCT05592444).

### Primary endpoint and power analysis

The primary endpoint was VA from a region of interest (ROI) placed in the PT at 40 keV.

Power analysis was based on an expected 300 Hounsfield units (HU) mean attenuation in the PT, equal in the groups, a 55 HU standard deviation and a 20 HU superiority margin. Power (1-β) = 0.8 and type I error rate, α = 5%. The calculated sample size was 94 in each group.

### Randomisation

The principal investigator randomised patients to either one of two experimental groups or the control group, with a 1:1:1 ratio, using the RANDBETWEEN (1,2,3) function in Microsoft Excel (Microsoft Corporation), and sealed the randomisation codes in sequentially numbered envelopes. Enrolment and allocation were performed by radiographers in the CT laboratory. The patients and personnel assessing outcomes were blinded to the randomisation.

### Patient population

Between 23 November 2020 and 12 October 2021, patients referred to CTPA at Akershus University Hospital were considered for enrolment in this single-centre parallel Randomised Controlled Trial (RCT). The inclusion criteria were eGFR > 30 mL/min/1.73 m^2^, clinical suspicion of PE, and peripheral venous catheter (PVC) ≤ 18 G. The exclusion criteria were contraindication to iodinated CM, age < 18 years, pregnancy, or unwillingness to participate for any reason.

### Imaging protocol

We used a bodyweight-adapted CM volume of iohexol 350 mgI/mL or 140 mgI/mL (Omnipaque 350, or 140, GE Healthcare). The control group A (dilution) received 0.4 mL/kg and CM was diluted 1:1 with saline, while the experimental group B (low flow) received 0.4 mL/kg with low flow and the experimental group C (low concentration) received 1 mL/kg with low concentration (140 mgI/mL). This allowed identical injection time in all groups.

The CM was administered using a power injector (CT Exprès TM 4D, Bracco Injeneering SA), followed by a 35 mL saline flush (not included in the injection time). We used an automatic bolus tracker with a ROI in the PT, 130 HU trigger point, and scan after 5 s. The maximum injected volume was limited to Group A: 72 mL (12.6 g), Group B: 36 mL (12.6 g) and Group C: 90 mL (12.6 g). Scans were performed supine, feet-first on a Philips IQon spectral detector scanner (Philips Healthcare). The scan parameters were tube voltage: 120 kVp, collimation: 64 × 0.625 mm, rotation time: 0.27 s, pitch: 1.2, matrix: 512 × 512, slice thickness: 0.9 mm, increment: 0.45 mm. Automatic tube current (DoseRight 3D-DOM, Philips Healthcare) was enabled, and the Dose Right Index was set at 18.

VMI were reconstructed in 40 and 55 keV with a spectral reconstruction algorithm: spectral B, denoising level 3 on an IntelliSpace Portal 9.0 workstation (Philips Healthcare). Images were assessed on 1 mm slice axial images.

### Secondary endpoints

Secondary endpoints were noise, signal-to-noise ratio (SNR), and subjective examination quality (SEQ) in the PT at 40 keV and objective examination quality (OEQ) and SEQ across all anatomical levels at 40 and 55 keV.

#### Objective examination quality

One radiographer performed the analysis of OEQ. In addition to the PT, ROIs were manually placed bilaterally in

- the interlobar arteries (ILA)
- the posterior basal segmental arteries (PBSA)

Each ROI was drawn as large as possible without including the arterial wall. ROIs were copied and pasted in the VMI to ensure measurements from identical areas. We recorded VA (HU) and noise (standard deviation of the VA as an inverse measure of consistency of attenuation). SNR was calculated as follows: ${{\rm{SNR}}}=\frac{{{\rm{VA}}}}{{{\rm{noise}}}}$. We defined VA < 211 HU as non-diagnostic OEQ [21].

#### Subjective examination quality

**Fig. 1:**
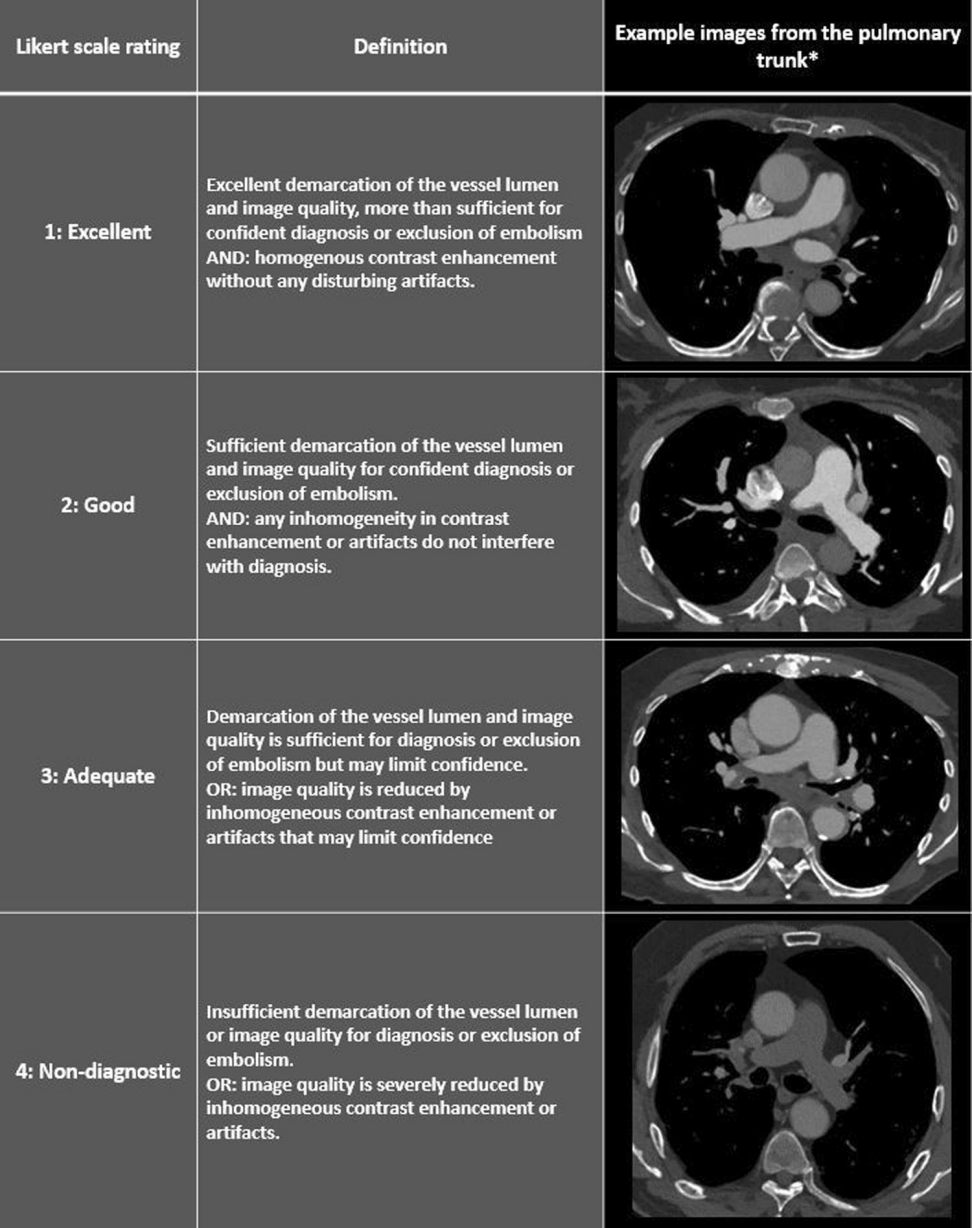
Likert rating scale for subjective examination quality with image examples. All images are 55 keV reconstructions shown in window level: 257 HU and window width: 1072 HU

SEQ was independently rated by one thoracic radiologist and one vascular interventional radiologist on 40 and 55 keV in the PT and bilaterally in the ILA and PBSA. The raters also registered the presence of PE, and in cases of occlusion, SEQ was not assessed at that anatomical level. A 4-point rating scale was used, as follows: 1: Excellent, 2: good, 3: adequate, 4: non-diagnostic, and dichotomised with ratings 1 through 3 considered diagnostic quality (Fig. 1). Disagreements on whether an examination had diagnostic quality were resolved by an independent rating by a third vascular interventional radiologist. The resolved rating was the median of the three ratings. In all other cases, the median of the two ratings was used for statistical analysis.

### Radiation dose estimation

The dose length product (DLP) and volume CT dose index (CTDIvol) was recorded for each patient, and the effective radiation dose (ED) was calculated by multiplying DLP by a conversion coefficient k for the chest: ED = DLP × k, k = 0.0145 mSv/mGycm [22].

### Statistical analysis

#### Primary endpoint

The hypothesis test for the primary endpoint was a pairwise *t*-test on the non-transformed attenuation in the PT at 40 keV between the randomisation groups, with the Holm method for multiple testing adjustment [23]. Cohen’s D effect sizes were reported.

#### Secondary endpoints

##### Secondary analyses in the PT at 40 keV:

Secondary OEQ outcomes in the PT were analysed with pairwise *t*-test on noise and SNR data, and effect sizes reported as Cohens D.

Secondary SEQ outcomes in the PT were tested with a non-parametric pairwise Wilcoxon rank-sum test with continuity correction, and effect sizes reported as rank-biserial coefficients.

### Sensitivity analysis

A sensitivity analysis was performed of the assumptions of the *t*-test on VA, noise, and SNR data from the PT. A pairwise *t*-test was performed on log-transformed data (to achieve approximate normal distribution), and effect sizes were reported as Cohens D. Further, differences in the distribution of VA, noise, and SNR in the PT were tested with a non-parametric pairwise Wilcoxon rank-sum test with continuity correction, and effect sizes reported as rank-biserial coefficients.

Correction for multiple testing was done with the Holm method [23].

### Analysis across anatomical levels and VMI levels

VA, noise, and SNR were analysed at every anatomical level and both VMI levels as dependent variables in multivariate linear mixed-effects regression models with Gaussian likelihood after log-transformation. Included random effects were a constant mean value for each patient, and fixed effects were randomisation group, age, sex, CTDI-vol, VMI level, anatomical level, and presence of PE. SEQ was analysed using a mixed-effects ordinal regression framework, including the same covariate dependence as the mean VA model, plus random mean effects included for each rater. Effect sizes were reported as regression coefficients (β) for standardised data and β > 0.5 was classified as large, β 0.1–0.5 as medium and β < 0.1 as small.

### Descriptive statistics

Patient and scan characteristics were reported as mean (SD) and proportions. Median and interquartile range of VA, noise, and SNR and proportions of non-diagnostic OEQ and SEQ are reported per VMI level and anatomical level. Interrater agreement for SEQ is reported as absolute agreement and weighted Fleiss kappa for the entire scale and Cohen’s kappa for the dichotomised scale. Kappa-values were interpreted according to Landis and Koch [24].

Statistical analyses were performed using Statistical Package for the Social Sciences version 29.0 (IBM) and R (version 4.3.2) using the lmerTest and ordinal packages [25–27].

## Results

### Inclusion and patient characteristics

**Fig. 2:**
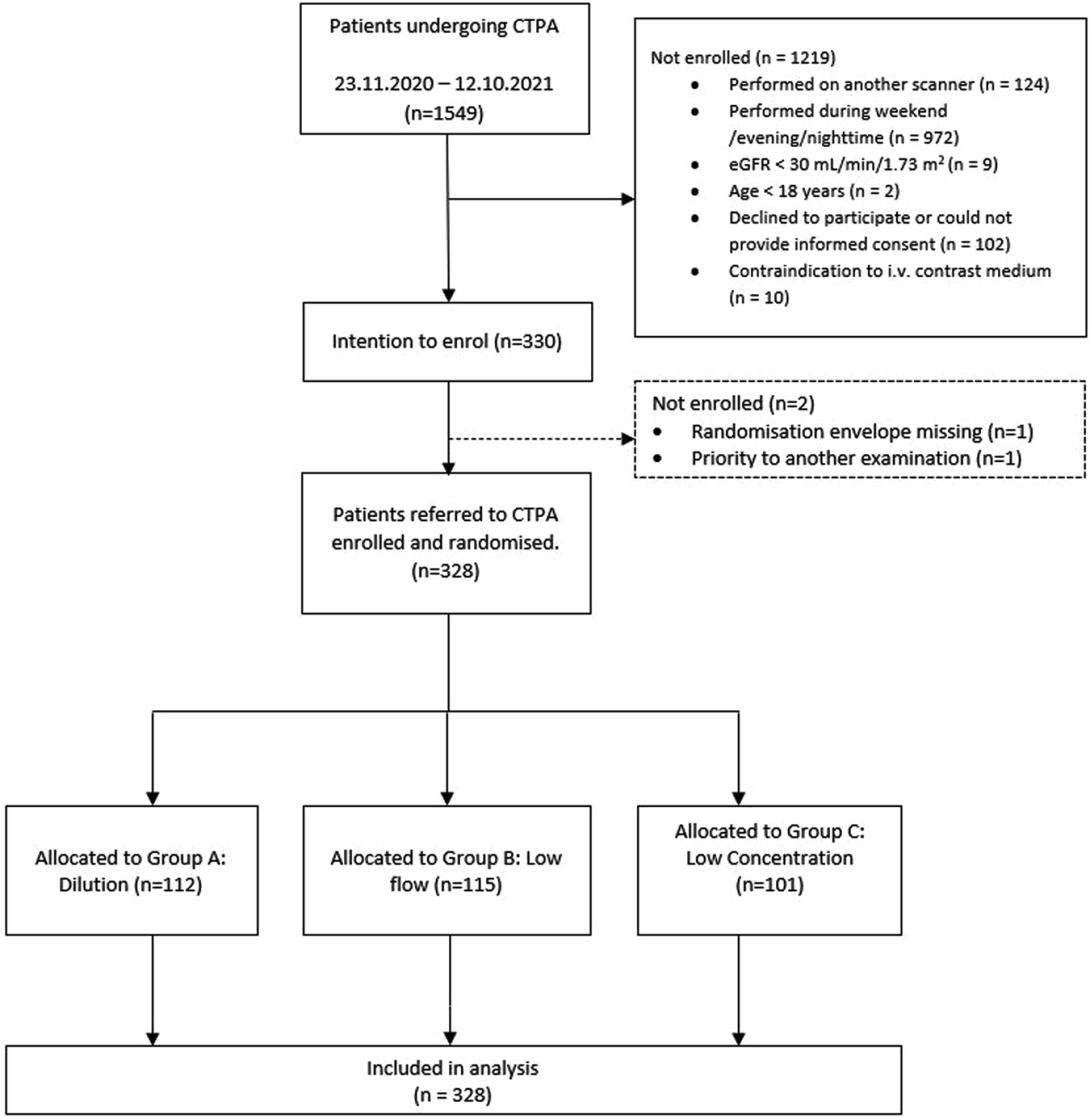
Flowchart of enrolment and randomisation

During the inclusion period, 1549 CTPAs were performed. Among these, we intended to enrol 330 patients. One randomisation envelope went missing, and one patient was reassigned to a different CT examination after randomisation. Hence, 328 patients were included in the analysis. Enrolment and inclusion are shown in Fig. 2.

**Fig. 3:**
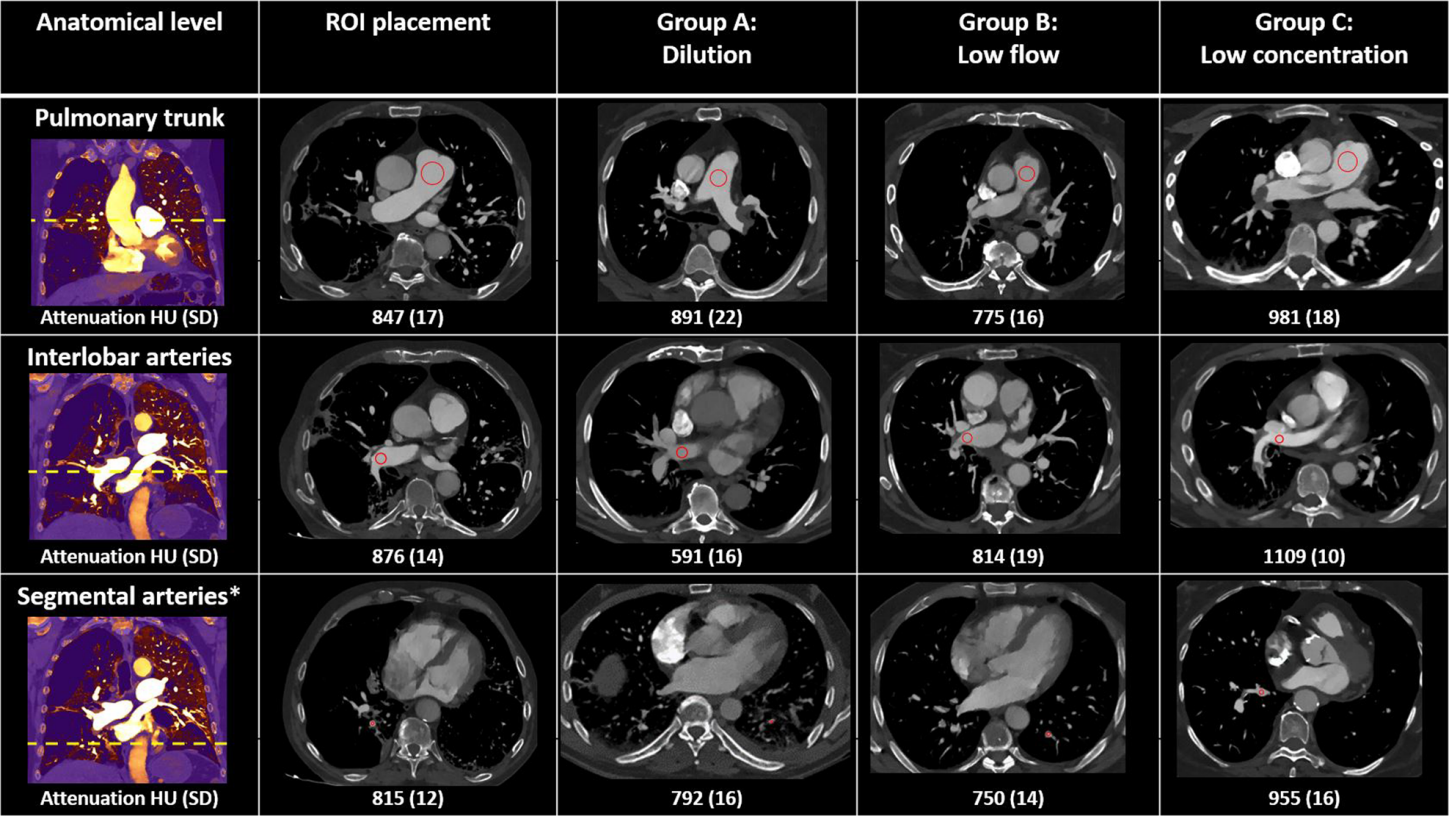
Illustration of correct ROI placement and pulmonary embolisms. The two columns to the left show correct ROI placement for the acquisition of vascular attenuation and noise measurements from the three anatomical levels in the study (pulmonary trunk, interlobar arteries and posterior basal segmental arteries). The three columns to the right show examples of pulmonary embolisms in approximately the same anatomical levels in the three randomisation groups. The presented attenuation and noise values from the randomisation groups were measured in ROIs as shown (red circles) but are not the actual measurements recorded in the study (as the pulmonary embolisms are not all in the exact same anatomical level). All images are 40 keV reconstructions. Window level: 547 HU and window width: 1922 HU. * Posterior basal segmental arteries

Patient and examination characteristics for the randomisation groups are shown in Table 1. Examples from the examinations showing pulmonary embolism in different anatomical levels are shown in Fig. 3.

**Table 1:** Patient and scan characteristics^a^

	Group A: Dilution (*n* = 112)	Group B: Low flow (*n* = 115)	Group C: Low concentration (*n* = 101)
Age (years)	63.2 (17.9)	62.7 (17.8)	65.6 (16.4)
Sex (male/female)	58/54	52/63	45/56
Weight (kg)	77.4 (19.1)	78.7 (21.5)	78.9 (18.4)
Pulmonary embolism detected^b^	21% (23/112)	19% (22/115)	19% (19/101)
Contrast volume (mL)	30.2 (7.3)	29.9 (5.9)	75.1 (13.6)
Saline volume (mL)	30.2 (7.3)^c^	N/A^c^	N/A^c^
Total iodine (g)	10.6 (2.6)	10.5 (2.1)	10.5 (1.9)
Flow rate (mL/s)	5.3 (1.0)	2.8 (0.6)	6.7 (1.2)
Injection time (s)	11.4 (2.0)	10.8 (0.6)	11.2 (0.3)
Iodine delivery rate (gI/s)	0.93 (0.17)	0.97 (0.20)	0.94 (0.17)
CTDI_vol_ (mGy)	10.3 (3.9)	10.7 (4.4)	10.8 (3.9)
DLP (mGy cm)	352.5 (134.4)	362.5 (145.3)	367.2 (129.3)
ED (mSv)^d^	5.1 (2.0)	5.3 (2.1)	5.3 (1.9)

Values are mean (SD) unless otherwise specified

*CTA* computed tomography angiography, *CTDI*_*vol*_ volumetric CT dose index, *DLP* dose length product, *ED* effective dose

^a^ Actual observed scan characteristics in each group

^b^ Percentage (proportion)

^c^ All patients received 35 mL of saline chaser after contrast injection

^d^ Conversion factor for peripheral CTA = 0.0145 mSv/mGycm

### Primary endpoint

There was no significant difference in the non-transformed attenuation in the PT between the randomisation groups: A vs. B (*p* = 0.96), A vs. C (*p* = 0.14), and B vs. C (*p* = 0.18) (Table 2).

**Table 2:** Analysis of differences between randomisation groups in attenuation (primary endpoint), noise of attenuation, signal-to-noise ratio and subjective examination quality in the pulmonary trunk at 40 keV

		*t*-test (pairwise)		
Randomisation groups		A vs B	A vs C	B vs C
**Attenuation**	**Effect size**	−0.0074	−0.27	−0.23
	***p*-value**	0.96	0.14	0.18
**Noise**	**Effect size**	−0.02	−0.05	−0.02
	***p*-value**	1.0	1.0	1.0
**Signal-to-noise ratio**	**Effect size**	−0.03	−0.23	−0.19
	***p*-value**	0.84	0.28	0.31

		Wilcoxon rank-sum (pairwise)		
**Subjective examination quality**	**Effect size**	0.05	0.07	0.03
	***p*-value**	0.67	0.44	0.67

Randomisation group A: Dilution, group B: Low flow, group C: Low concentration. Effect sizes were reported as Cohens D for *t*-tests and as rank-biserial coefficients for Wilcoxon rank-sum test

### Secondary endpoints

#### Secondary analyses in the PT

The hypothesis testing between the groups for the secondary endpoints in the PT is shown in Table 2.

The pairwise tests showed no significant differences in noise or SNR between the groups.

Pairwise Wilcoxon rank-sum test did not show any differences in distribution of SEQ between the groups.

#### Sensitivity analysis

The pairwise *t*-test after log-transformation did not show significant differences in VA, while a Wilcoxon rank-sum test showed significant differences in distribution between group C and the other groups (A vs C: *p* = 0.025 and B vs C: *p* = 0.013), but not between group A and B (*p* = 0.63). There were no differences in noise or SNR (Supplementary Table 1).

#### Secondary analyses of objective and subjective examination quality across anatomical levels

The distribution of OEQ and SEQ across groups, anatomical levels, and VMI levels are shown in Table 3. The multivariate mixed model regression analyses of associations between randomisation group, anatomical level, patient and examination factors and objective and subjective examination quality are shown in Table 4.

**Table 3:** Descriptive statistics for vascular attenuation, noise of attenuation, objective- and subjective examination quality by RCT groups with VMI at 40 and 55 keV from the truncus pulmonalis, interlobar arteries and segmental arteries

Anatomical level	Pulmonary trunk						Interlobar arteries						Segmental arteries				
VMI level	40 keV			55 keV			40 keV			55 keV			40 keV			55 keV	
**Objective examination quality**																	
**RCT-group**	**Attenuation**	**Noise**		**Attenuation**	**Noise**		**Attenuation**	**Noise**		**Attenuation**	**Noise**		**Attenuation**	**Noise**		**Attenuation**	**Noise**
**A**	762 (269)	19 (8)		416 (140)	15 (6)		739 (261)	16 (8)		408 (135)	14 (6)		697 (259)	18 (12)		380 (132)	14 (9)
**B**	735 (297)	18 (7)		399 (152)	15 (5)		732 (293)	18 (9)		402 (157)	15 (6)		695 (259)	19 (19)		379 (139)	16 (12)
**C**	835 (232)	19 (7)		452 (118)	15 (4)		825 (223)	17 (8)		449 (111)	14 (5)		768 (211)	17 (14)		417 (109)	14 (11)
**Proportion of non-diagnostic objective examination quality**^**a**^																	
**A**	3/112			4/112			7/223^b^			9/223^b^			5/223^b^			10/223^b^	
**B**	4/115			7/115			5/230			9/230			3/230			13/230	
**C**	2/101			2/101			2/202			4/202			3/202			5/202	
**Subjective examination quality**^**c**^																	
**A**	1/112 [1.5]			2/112 [1.5]			0 [1.0]			2/223 [1.5]			5/223 [1.5]			5/223 [1.5]	
**B**	0 [1.5]			0 [1.5]			0 [1.0]			0 [1.0]			0 [1.0]			0 [1.0]	
**C**	0 [1.5]			0 [1.5]			0 [1.0]			0 [1.0]			0 [1.5]			0 [1.5]	

For attenuation and noise of attenuation numbers are Median (IQR)

*IQR* interquartile range, *keV* kiloelectron volt, *RCT* randomised control trial

^a^ For objective examination quality numbers are proportions of non-diagnostic quality, i.e., vascular attenuation < 211 HU

^b^ One patient in this group had a previous pulmectomy, resulting in one less measurement in the interlobar and segmental arteries

^c^ For subjective examination quality numbers are proportions of non-diagnostic rating, i.e., Likert 4 and [median score]

**Table 4:** Multivariate mixed model regression analyses of associations between randomisation group, anatomical level, patient and examination factors and objective- and subjective examination quality

		Randomisation group^a^		Anatomical level^b^		Sex^c^	Age	Pulmonary embolism	CTDIvol	VMI level^d^
		Group B	Group C	Interlobar arteries	Segmental arteries					55 keV
**Attenuation**^e^	**e.s.**	0.019	0.11	−0.0062	−0.050	0.018	0.036	0.0096	0.015	−0.57
	**s.e.**	0.042	0.043	0.010	0.011	0.037	0.018	0.011	0.019	0.0069
	***p***-**value**	0.7	**0.01**	0.6	**≪ 0.001**	0.6	**0.04**	0.4	0.4	**≪ 0.001**
**Noise**^e^	**e.s.**	0.045	0.0086	−0.066	0.049	0.088	0.0015	0.033	0.033	−0.22
	**s.e.**	0.033	0.035	0.019	0.020	0.030	0.00081	0.018	0.0037	0.014
	***p***-**value**	0.2	0.8	**≪ 0.001**	**0.01**	**0.003**	0.06	0.07	**≪ 0.001**	**≪ 0.001**
**Signal-to-noise ratio**^e^	**e.s.**	−0.024	0.096	0.070	−0.10	−0.069	0.0092	−0.020	−0.12	−0.35
	**s.e.**	0.041	0.043	0.022	0.023	0.037	0.017	0.021	0.019	0.016
	***p***-**value**	0.56	**0.025**	**0.0019**	**≪ 0.001**	0.062	0.60	0.34	**≪ 0.001**	**≪ 0.001**
**Subjective examination quality**^f^	**e.s.**^g^	−0.26	−0.21	−1.4	−0.71	0.15	−0.15	0.79	0.69	0.29
	**s.e.**	0.21	0.22	0.087	0.082	0.19	0.089	0.33	0.094	0.064
	***p***-**value**	0.2	0.3	**≪ 0.001**	**≪ 0.001**	0.4	0.08	**0.02**	**≪ 0.001**	**≪ 0.001**

Effect sizes (e.s.), standard errors (s.e.), and *p*-values are all derived from the corresponding regression coefficients

Group A (dilution), group B (low flow), group C (low concentration)

*VMI* virtual monoenergetic imaging, *keV* kiloelectron Volt

^a^ Effects are compared to group A

^b^ Effects are compared to pulmonary trunk

^c^ Male as reference

^d^ Effects are compared to 40 keV

^e^ Multivariate linear mixed-effects regression models with Gaussian likelihood after log-transformation

^f^ Multivariate ordinal mixed-effects regression model

^g^ Negative effect sizes indicate an improved subjective examination quality

Bold values represent statistical significance *p*-values

The analysis showed a significantly higher VA (*p* = 0.01) and SNR (*p* = 0.025) in group C (low concentration) compared to the reference (group A: dilution), with medium and small effect sizes, respectively. There were no differences in noise between the groups.

To aid interpretation exponential transformations of the effect sizes show approximate 2% and 12% increases in VA in groups B and C, respectively, compared to group A. With regard to noise, the corresponding effects were 5% and 1% increases in groups B and C, respectively, and SNR showed a 2% decrease in group B and a 10% increase in group C—all compared to group A.

The ratings and the interrater agreement from the two raters are shown in Table 5. There was a slight interrater agreement for the 4-point scale with a kappa of 0.18 both in the PT and across all anatomical levels, with absolute agreement of 33% and 52%, respectively. There was fair interrater agreement on the dichotomous scale in the PT (kappa: 0.33) and slight across all anatomical levels (kappa: 0.03), with absolute agreement of 99% and 96%, respectively. The median resolved rating of SEQ was 1.5 in the PT and 1 across all anatomical levels. The rate of non-diagnostic SEQ was 0.5%. Table 4 shows no differences in SEQ between the groups, with a 0.2 decrease in odds ratio for a positive rating in groups B and C compared to group A.

**Table 5:** Subjective examination quality ratings from the two raters in the pulmonary trunk at 40 keV and for all data^a^

Pulmonary trunk at 40 keV		Rater 1			
		1	2	3	4
**Rater 2**	**1**	89	186	9	0
	**2**	3	18	8	0
	**3**	1	8	1	0
	**4**	1	1	2	1
Weighted Kappa: 0.18, Absolute agreement: 33% Dichotomous Kappa^b^: 0.33, Absolute agreement: 99%					

All data^a^		Rater 1			
		1	2	3	4
**Rater 2**	**1**	1504	1180	82	6
	**2**	51	144	25	0
	**3**	21	31	7	0
	**4**	29	54	22	2
Weighted Kappa: 0.18, Absolute agreement: 52% Dichotomous Kappa^b^: 0.03, Absolute agreement: 96%					

^a^ Both 40 and 50 keV and all three anatomical levels

^b^ Ratings 1 through 3: diagnostic and rating 4: non-diagnostic

All numbers are *n*

*keV* kiloelectron Volt

From 3276 measurements of VA across groups, anatomical levels, and VMI levels 97 (3%) showed non-diagnostic OEQ, and the proportion ranged from 1% to 6.1% across groups, anatomical levels, and VMI levels.

Out of 3276 subjective ratings across groups, VMI levels, and anatomical levels, 15 (0.5%) were rated non-diagnostic. These were all in group A, and 10 were at the segmental artery level.

## Discussion

In this RCT there was no difference between the groups, A: dilution, B: low flow, and C: low concentration, in attenuation in the PT. This means that all three strategies may still be used in clinical practice, which makes implementation of low CM dose protocols easier.

However, group C showed 73 and 100 HU higher mean attenuation in the PT than group A and B (Table 3), and a sensitivity analysis (Supplementary Table 1) showed a difference in distribution in Group C compared to A and B (*p* = 0.025 and 0.013). Group C also showed higher VA when analysing data across all anatomical levels, corresponding to an approximate increase of 12% compared to group A. These results should be interpreted cautiously since they were secondary endpoints but align with previously reported data showing higher peak vascular attenuation with lower concentration CM [28, 29].

The latter result may, in part, be due to the higher number of measurements, five per patient, giving increased statistical power when using data across all anatomical levels. Acquiring measurements at several anatomical levels proved to be relevant since, compared to the PT, the mean attenuation in the segmental arteries was 40–67 HU lower at 40 keV (and 20–36 HU lower at 55 keV) (Table 3). This effect was statistically significant (*p* ≪ 0.001), although with a small effect size (Table 4).

### Non-diagnostic OEQ and SEQ

The threshold of 211 HU as a criterion for diagnostic OEQ was based on a study calculating the minimum pulmonary arterial enhancement for differentiating intraluminal CM from acute and chronic PE to be 93 and 211 HU, respectively [21, 30] Such thresholds vary in the literature from 180 to 250 and are usually based on clinical experience [31, 32]. The percentage of non-diagnostic OEQ and SEQ were 3% and 0.5%, respectively. This is in the lower range of the previously reported 3–5% rate of inconclusive examinations [1, 33, 34].

For the SEQ, the slight to fair kappa-agreement between raters may in part be due to their different areas of specialty, but also underlines the subjective nature of the scale [24]. However, the combination of relatively low kappa-values compared to higher absolute agreement is a frequent finding when the distribution of ratings is uneven (the kappa paradox), as in this case, with very few low ratings compared to high [35]. SEQ was rated as non-diagnostic in four examinations, all in group A, but there was no significant difference between groups. A great majority of examinations with non-diagnostic OEQ were rated as diagnostic SEQ, showing that raters in this study assessed that they could make diagnostic interpretations of examinations with VA below the objective threshold.

### CM-reduction rationale and strategies

It is the responsibility of healthcare providers to minimise patient and environmental exposure to CM and thus, reduce patient complications, hospital costs, and pollution of the environment.

This is especially important for patients with impaired kidney function, as the risk of post-contrast acute kidney injury increases with the applied volume of CM [12].

The previously demonstrated augmentation of the attenuation of iodine with DECT allows a substantial reduction of CM dosage [3, 4, 36, 37]. Weight-based dosage, which was applied in all three groups, may also reduce CM dose [2]. Other studies have found that individually tailored CM in CTPA can provide increased image quality [32, 38]. Keeping injection time constant and reducing iodine delivery rate (IDR) is a robust method to avoid changes in scan timing when reducing the CM dose. In this study IDR reduction was accomplished by CM-dilution, lowering flow or lowering iodine concentration. For all three strategies, the injection time (11 s) was constant.

### Advantages and drawbacks of the three strategies

At body temperature, the viscosity of the low concentration CM used in group C (140 mg I/mL) is 1.5 mPa s, which is much lower than the 10.6 mPa s viscosity of the high concentration CM used in group A and B (350 mg I/mL) [39].

Lower viscosity facilitates vascular distribution and a more homogenous bolus and improves enhancement in smaller vessels, which is particularly important in angiography and may account for the improved attenuation observed in group C [28, 40, 41].

The application of dilution injection, mixing CM with saline (as in group A) is readily applicable in clinical practice and the standard injection protocol at our institution [7, 42]. However, even given successful mixing of 350 mg I/mL CM with equal parts saline in the injector, the viscosity is estimated to approximately 1.9 mPa s, which is roughly 27% higher than for the low concentration CM [39].

Although we have shown possible advantages to the low concentration strategy in CTPA, a higher concentration (≥ 350 mg I/mL) CM is recommended for many other examinations [43]. This may complicate clinical implementation since changing CM concentration in the power injector between examinations may be cumbersome. In larger or more specialised units, it may be feasible to run low concentration CM protocols on one lab and high concentration CM protocols on another. Further, this issue may be remedied by new injectors designed for multiple CM bottles allowing CT labs to have different concentrations of CM in the power injector simultaneously.

The mean flow rate was the highest in group C, which reportedly raises concerns regarding poor PVC or fragile veins although, another study reports high flow rates to be feasible without complications, suggesting it is no drawback in clinical practice [44, 45].

The use of low flow (group B) may benefit patients with small PVC or fragile veins and provides sufficient enhancement [44]. Park et al showed, in an extensive patient cohort, that reducing the CM dosage by lowering the injection rate notably decreases the incidence of immediate CM hypersensitivity reactions [11]. In our study, there were no serious immediate CM hypersensitivity reactions in any of the groups, but we did not systematically record moderate or mild reactions.

### Future perspectives

Photon-counting detector CT (PCD-CT) may provide more accurate data on x-ray intensity and energy levels, thus enabling more precise iodine quantification. Combined with VMI, this may facilitate further reductions in CM while maintaining high image quality [46]. Recent studies demonstrate the potential for CM reduction in patients undergoing CTPA with PCD-CT [47, 48]. The three strategies for reducing CM presented in this study are most likely transferable to PCD-CT.

### Limitations

Our study focused exclusively on CTPA and used one CT scanner from a specific vendor. While this may affect generalisability, all three methods may be applied to various vascular examinations and any scanner, and it is unlikely that the scanner impacted the differences between the groups significantly. We only used a power injector from one vendor, and the results might have been different with a different injector. The findings of this study may have limited validity for examinations of parenchymal organs with longer scan delay.

### Strength

To the best of our knowledge, this RCT is the first study comparing multiple methods of contrast administration in CTPA with low dose CM in clinical practice.

The study was performed with an unselected cohort of patients referred for CTPA and did not exclude patients with cardiac failure or patients with larger body habitus. Hence, our results are valid for clinical imaging and larger patients. The detection rate of pulmonary embolism was 19.5%, which is comparable to other studies, and indicates that the cohort is representative [49, 50].

## Conclusion

There was no difference in the attenuation in the PT between the dilution, low flow, and low concentration groups. However, the low concentration group showed higher attenuation in the pulmonary arteries when all anatomical levels were assessed.

## Supplementary information

ELECTRONIC SUPPLEMENTARY MATERIAL

## Abbreviations

CM: Contrast medium
CTDIvol: Volume CT dose index
CTPA: CT pulmonary angiography
DE: Dual-energy
DECT: Dual-energy CT
DLP: Dose length product
ED: Effective dose
eGFR: Estimated glomerular filtration rate
HU: Hounsfield units
IDR: Iodine delivery rate
ILA: Interlobar arteries
keV: Kiloelectron volt
kVp: Kilovolt peak
Noise: Image noise
OEQ: Objective examination quality
PBSA: Posterior basal segmental arteries
PE: Pulmonary embolism
PT: Pulmonary trunk
PVC: Peripheral venous catheter
RCT: Randomised controlled trial
ROI: Region of interest
SD: Standard deviation
SEQ: Subjective examination quality
VA: Vascular attenuation
VMI: Virtual monoenergetic images
