# Supplementary material for: A randomised controlled trial comparing three clinical administration strategies in spectral detector CT pulmonary angiography with low contrast medium dose
Source: Eur Radiol. 2025 Feb 19;35(8):4649–60. doi: 10.1007/s00330-025-11420-8 (PMC12226604; doi:10.1007/s00330-025-11420-8)
Supplement: Supplementary file 1 — ELECTRONIC SUPPLEMENTARY MATERIAL [file 330_2025_11420_MOESM1_ESM.pdf]

A Randomised Controlled Trial comparing three clinical administration strategies in spectral detector CT Pulmonary Angiography with low contrast medium dose

ELECTRONIC SUPPLEMENTARY MATERIAL

Supplementary table 1: Sensitivity analysis of differences between randomisation groups in attenuation, noise of attenuation, and signal to noise ratio in the pulmonary trunk at 40 keV.

**Statistical test**		**t-test (pairwise) log-transformed data**			**Wilcoxon rank-sum (pairwise)**		
**Randomisation groups**		**A vs B**	**A vs C**	**B vs C**	**A vs B**	**A vs C**	**B vs C**
**Attenuation**	**Effect size**	0.07	-0.16	-0.21	0.04	-0.20	-0.22
	***p*-value**	0.62	0.47	0.37	0.63	**0.025**	**0.013**
**Noise**	**Effect size**	0.05	-0.06	-0.10	0.07	-0.03	-0.11
	***p*-value**	1.0	1.0	1.0	0.69	0.69	0.48
**Signal to noise ratio**	**Effect size**	0.03	-0.12	-0.14	-0.006	-0.14	-0.12
	***p*-value**	0.95	0.95	0.95	0.94	0.26	0.26

Randomisation group A: Dilution, group B: Low flow, group C: Low concentration.
Effect sizes were reported as Cohens D for t-tests and as rank-biserial coefficients for Wilcoxon rank-sum test
